# Integrating Machine Learning and Follow-Up Variables to Improve Early Detection of Hepatocellular Carcinoma in Tyrosinemia Type 1: A Multicenter Study

**DOI:** 10.3390/ijms26083839

**Published:** 2025-04-18

**Authors:** Karen Fuenzalida, María Jesús Leal-Witt, Alejandro Acevedo, Manuel Muñoz, Camila Gudenschwager, Carolina Arias, Juan Francisco Cabello, Giancarlo La Marca, Cristiano Rizzo, Andrea Pietrobattista, Marco Spada, Carlo Dionisi-Vici, Verónica Cornejo

**Affiliations:** 1 Laboratory of Genetic and Metabolic Diseases, Institute of Nutrition and Food Technology INTA, University of Chile, Av. El Libano 5524, Santiago 7830490, Chile; mj.leal@inta.uchile.cl (M.J.L.-W.); a.acevedo.aracena@gmail.com (A.A.); manuel.munoz.g@ug.uchile.cl (M.M.); camila.gudenschwager@gmail.com (C.G.); carias@inta.uchile.cl (C.A.); jfcabello@inta.uchile.cl (J.F.C.); vcornejo@inta.uchile.cl (V.C.); 2 Meyer Children’s Hospital IRCCS, Viale Gaetano Pieraccini, 24, 50139 Florence, Italy; giancarlo.lamarca@meyer.it; 3 Department of Experimental and Clinical Biomedical Sciences, University of Florence, Largo Brambilla, 3, 50134 Florence, Italy; 4 Division of Metabolic Diseases and Hepatology, Ospedale Pediatrico Bambino Gesù IRCCS, 00165 Rome, Italy; cristiano.rizzo@opbg.net (C.R.); andrea.pietrobattista@opbg.net (A.P.); carlo.dionisivici@opbg.net (C.D.-V.); 5 Division of Abdominal Transplantation, Hepato-Bilio-Pancreatic Surgery Unit, Ospedale Pediatrico Bambino Gesù IRCCS, 00165 Rome, Italy; marco.spada@opbg.net

**Keywords:** inborn errors of metabolism, artificial intelligence, hepatocellular carcinoma, alpha-fetoprotein, liver biomarkers, hereditary tyrosinemia type 1

## Abstract

Hepatocellular carcinoma (HCC) is a major complication of tyrosinemia type 1 (HT-1), an inborn error of metabolism affecting tyrosine catabolism. The risk of HCC is higher in late diagnoses despite treatment. Alpha-fetoprotein (AFP) is widely used to detect liver cancer but has limitations in early-stage HCC detection. This study aimed to implement a machine-learning (ML) approach to identify the most relevant laboratory variables to predict AFP alteration using constrained multidimensional data from Chilean and Italian HT-1 cohorts. A longitudinal retrospective study analyzed 219 records from 35 HT-1 patients, including 8 with HCC and 5 diagnosed through newborn screening. The dataset contained biochemical and demographic variables that were analyzed using the eXtreme Gradient Boosting algorithm, which was trained to predict abnormal AFP levels (>5 ng/mL). Four key variables emerged as significant predictors: alanine transaminase (ALT), alkaline phosphatase, age at diagnosis, and current age. ALT emerged as the most promising indicator of AFP alteration, potentially preceding AFP level changes and improving HCC detection specificity at a cut-off value of 29 UI/L (AUROC = 0.73). Despite limited data from this rare disease, the ML approach successfully analyzed follow-up biomarkers, identifying ALT as an early predictor of AFP elevation and a potential biomarker for HCC progression.

## 1. Introduction

Tyrosinemia type 1 (HT-1, OMIM 276700) is an inborn error of metabolism caused by a defect in the enzyme fumarylacetoacetate hydrolase (FAH) involved in the final step of tyrosine degradation with an estimated prevalence of 1 in 100,000 to 120,000 births worldwide, with higher prevalence in Quebec, Canada (~1 in 16,000 births). This pathophysiological condition arises from the accumulation of toxic intermediate metabolites in hepatic and renal tissues. Untreated affected individuals typically exhibit hepatorenal manifestations at an early age, associated with a high risk of developing hepatocellular carcinoma (HCC) [1,2]. Other manifestations include renal Fanconi syndrome, hypophosphatemic rickets, developmental delay, and porphyria-like neurological crises. Diagnosing HT-1 relies on identifying elevated levels of toxic metabolite succinylacetone (SUAC) in blood or urine, either when acute clinical symptoms appear or through newborn screening. In Chile, the detection of HT-1 typically occurs at an advanced stage, when patients already present disease-associated symptoms, since HT-1 is not included in the National Newborn Screening Program. In most cases, there is a substantial increase in the tumor biomarker alpha-fetoprotein (AFP) and mainly acute hepatic failure. In this context, newborn screening for HT-1 and early initiation of treatment is pivotal to preventing the onset of symptoms and reducing the risk of liver malignancy. Therefore, early screening leads to an almost complete suppression of hepatic and renal diseases throughout life. The treatment of HT-1 patients consists of a combined pharmacological and nutritional regimen, including nitisinone (2-(2-nitro-4-trifluoromethylbenzoyl)-cyclohexane-1,3-dione, NTBC) and a tyrosine- and phenylalanine-restricted diet. NTBC, developed initially as a herbicidal compound, was later repurposed as a highly effective treatment for HT-1 [2,3,4,5,6]. Once NTBC treatment starts, SUAC levels become almost undetectable in most cases, and AFP decreases gradually until reaching normal levels [7,8]. Despite the remarkable efficacy of NTBC, a subset of late-treated patients will require liver transplantation at some point in their lives due to the development of HCC [9,10,11]. Risk factors for HCC in these patients include delayed NTBC therapy initiation, suboptimal NTBC response, cirrhosis, and the slow decline of AFP levels after starting NTBC therapy [12,13]. Currently, AFP levels and liver imaging are the main lines for identifying HCC and its progression; however, recent reports indicate that elevated AFP levels may not be sensitive enough to detect very early HCC stages [14,15] and sudden liver nodule formation [16]. Some patients develop HCC after an acute increase in AFP levels [16], while others have HCC despite normal AFP levels [13,17,18]. No routine biomarkers can predict this complication.

Each outpatient visit generates a dataset of categorical and continuous variables and a temporal component. Consequently, it is complicated to analyze the relationships between variables and evaluate their potential as biomarkers. Machine learning (ML)-based strategies have been applied to congenital metabolic disorders for the past 10 years, primarily for diagnostics improvement in newborn screenings [19,20,21,22,23]. In such cases, datasets comprising many features, often in the order of thousands, are employed. In contrast, in the case of HT-1, training an algorithm with a dataset obtained from follow-up visits to predict disease prognosis or identify patient subclasses can be challenging due to the limited number of patients and the small number of routinely scanned variables. Nevertheless, some strategies have been devised to overcome this challenge in recent years [24,25]. We recently developed an ML multi-model approach based on data from follow-up visits to predict the risk of developing insulin resistance in adult patients with phenylketonuria [25]. Here, we identified parameters contributing to insulin resistance in phenylketonuria, with phenylalanine levels being the second most crucial variable after body mass index, a known risk factor [26,27]. Multiple independent training rounds generated models with varying performance levels, allowing us to focus on high-performing models for robust phenotype generalization [25]. This ML approach proved helpful in studying pathologies with a scarce number of patients and high data dimensionality, such as inborn errors of metabolism.

In this work, we applied a multi-model ML approach to integrate biochemical and demographic data from three HT-1 patient cohorts from Chile and Italy, with varying diagnosis times and NTBC exposure. This approach generated interpretable predictive models capable of identifying the importance of routine laboratory parameters in predicting abnormal levels of AFP. Understanding the significance of these biomarkers and their relationship with AFP could potentially improve the early detection of hepatocellular carcinoma (HCC) progression in HT-1 patients.

## 2. Results

### 2.1. Patient Cohort Characterization

This retrospective study involved the analysis of follow-up data from thirty-five HT-1 patients across three metabolic centers, including five patients with neonatal screening from Florence, eight patients who developed hepatocellular carcinoma and underwent liver transplantation, two cases from Chile, and six cases from Rome. Forty-eight percent of the individuals were female, and the median age of patients was 7.7 years old in Chile, 12.3 years for Rome, and 4.5 years for Florence (Table S1). The age at the time of diagnosis, a crucial factor in the risk of hepatocellular carcinoma (HC), presented a median for the cohorts from Chile, Rome, and Florence of 9 months (min–max: 1–63), 20 months (min–max: 6–48), and 0.2 months (min–max: 0.2–0.33), respectively, exhibiting statistical differences (*p* < 0.01) between the Florence cohort and the Chilean and Roman cohorts (Table S1). All patients evaluated at Meyer’s Hospital in Florence were diagnosed within the first six to nine days after birth through newborn screening. In contrast, most patients from Rome and Chile were diagnosed later in their development based on symptom presentation and clinical suspicion. Regarding AFP levels, a biomarker of HCC, the Chilean cohort displayed a median of 9.6 ng/mL (min–max: 1.3–505), the Rome cohort a median of 39.7 ng/mL (min–max: 2.3–3467), and the Florence cohort a median of 2.2 ng/mL (min–max: 1.7–4.4). The highest median level observed in the Rome cohort is explained by six out of ten patients developing HCC, leading to a concomitant increase in AFP. The median levels of AFP and other variables, grouped by patients who developed or did not develop HCC in each cohort, are detailed in Table S2 (Rome and Chile cohorts).

**Figure 1:**
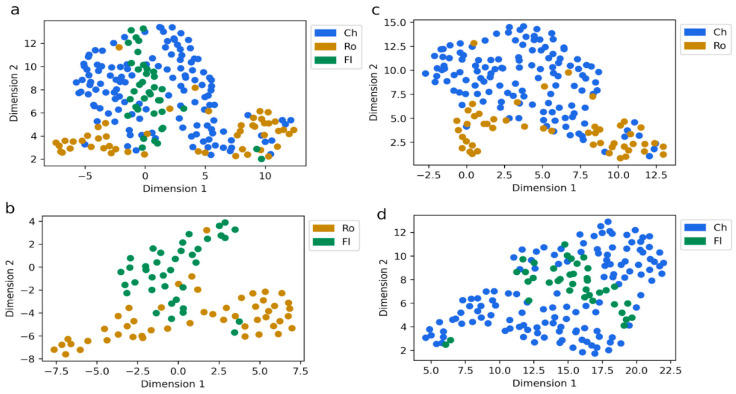
Dimensionality reduction across patient cohorts. (**a**) Analysis of patients from Chile (Ch), Rome (Ro), and Florence (Fl). (**b**) Analysis of Rome and Florence cohorts; (**c**) Chile and Rome cohorts; and (**d**) Chile and Florence cohorts. Each point corresponds to an individual patient. Dimensionality reduction was performed via Uniform Manifold Approximation and Projection.

We conducted a dimensionality reduction analysis to visualize clustering patterns in an interpretable two-dimensional space. In Figure 1, each dot represents an individual record of each patient, while different colors indicate their respective cohorts. Figure 1a shows the dimensionality reduction for the three cohorts, Chile (Ch), Rome (Ro), and Florence (Fl), where it can be observed that patients from the same cohort tend to cluster together. This pattern suggests that individuals within each cohort share some specific characteristics; however, there is a degree of overlap among the cohorts (Figure 1a). Furthermore, these patterns persisted when we analyzed the data by reducing dimensions for pairs of cohorts (as shown in Figure 1b–d). This consistency indicates cohort boundaries remain partially blurred rather than distinctly separated, demonstrating a complex interplay of attributes.

### 2.2. AFP Association with Follow-Up Variables

Considering the AFP values from all patients, we first set out to investigate whether AFP concentration exhibited significant correlations with any of the other analyzed variables. Table 1 lists the Spearman’s correlation (ρ) and *p*-values. Moderate significant correlations (ranging from ρ 0.53 to 0.45) were identified for transaminases and transferase in the following order: ALT > AST > GGT. The risk factor for HCC development, age at the time of diagnosis, presented a significant association with AFP (ρ 0.49, *p*-value < 0.0001). Other liver function biomarkers, such as prothrombin time, total bilirubin, and alkaline phosphatase, were significantly associated with AFP but weaker (ranging from ρ 0.29 to 0.208, *p*-value < 0.001). No correlation was found between AFP levels and metabolic (Met, Tyr, and Phe) or biochemical parameters (glycemia), nor between AFP and NTBC blood levels and NTBC and age at the time of control.

These results revealed which variables were most closely associated with AFP levels; however, we could not determine the influence of each biomarker or their interrelationships in predicting subtle abnormal changes in AFP levels. To further investigate the importance and contribution of these variables to the prediction of AFP alterations, we conducted an ML-based analysis across the three cohorts.

The entire dataset from the three HT-1 patient cohorts was used to correlate AFP levels with each variable. Statistical analysis used Spearman’s rank correlation coefficient (ρ) to evaluate linear relationships. Significant correlations (*p* < 0.05) are highlighted in red.

### 2.3. Relevance of Biochemical Variables as Features Related to AFP Levels

We hypothesized that fluctuations in certain laboratory variables could serve as early indicators of liver stress or progressive pathology in HT-1 patients. These changes may precede alterations in more definitive markers of HCC onset, such as AFP. We employed supervised ML-based analysis to further explore the importance of biochemical variables and age-related factors in classifying AFP levels as normal or elevated. To first define the AFP threshold, we analyzed the data from our HT-1 cohort, including patients who developed HCC and underwent liver transplantation (n = 8). We found a significant difference in median AFP values between the HCC and non-HCC groups (*p* < 0.0001). For patients who had not developed HCC, the median AFP value was 4.3 ng/mL (min: 1.1, max: 79.5, n = 190), while for the HCC patient group, the median was 61.1 ng/mL (min: 2.4, max: 13700, n = 41). Considering these findings and Choi et al.’s recent study [14] demonstrating improved early HCC detection using a 5 ng/mL AFP threshold in at-risk patients, we adopted this cut-off value. The machine learning analysis then explored variables most significantly related to the binarized AFP status (normal or altered).

**Figure A1:**
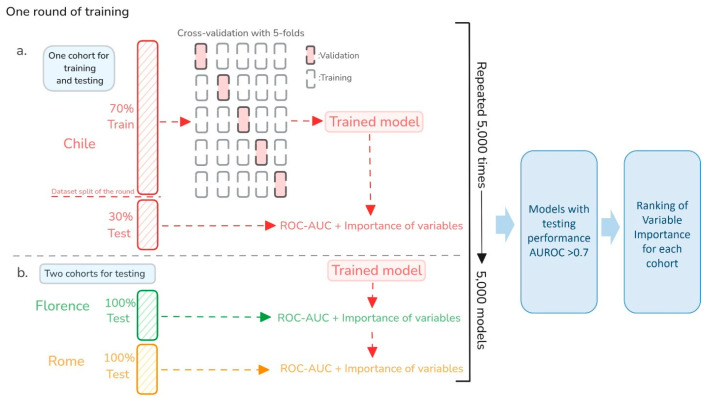
Model development workflow. Models were developed through training rounds using (**a**) the Chilean patient cohort for model training and internal testing and independent Italian cohorts from Rome and Florence for external testing (**b**). For the Chilean cohort, data were randomly partitioned into training (70%) and testing (30%) sets for each round, with new randomization performed between rounds to ensure robust cross-validation. Each training round generated a distinct model, allowing for the assessment of model stability and performance across different data partitions. Only the model with testing performance AUROC > 0.7 was considered for establishing the ranking of variable importance, which was calculated using the corrected importance (the product between the importance and the ROC-AUC averaged across all models).

We implemented a robust machine learning approach involving 5000 rounds of independent model training using bootstrapped datasets to mitigate challenges associated with limited data and high-dimensional features. Each ML model was trained exclusively using the Chilean cohort (70%), with a new randomized training set generated for each round. The test dataset remained independent throughout (30%), ensuring no records were used simultaneously for training and testing (See Section 4 and Figure A1). The cohorts from Rome and Florence served as external test datasets to assess the models’ generalizability. We evaluated the testing performance of each model using the receiver operating characteristic–area under the curve (ROC-AUC) metric, simultaneously analyzing the relative importance of each variable in predicting alpha-fetoprotein (AFP) status. This comprehensive methodology enabled us to generate diverse models, each capturing different nuanced aspects of the dataset while ensuring robust predictive performance.

**Figure 2:**
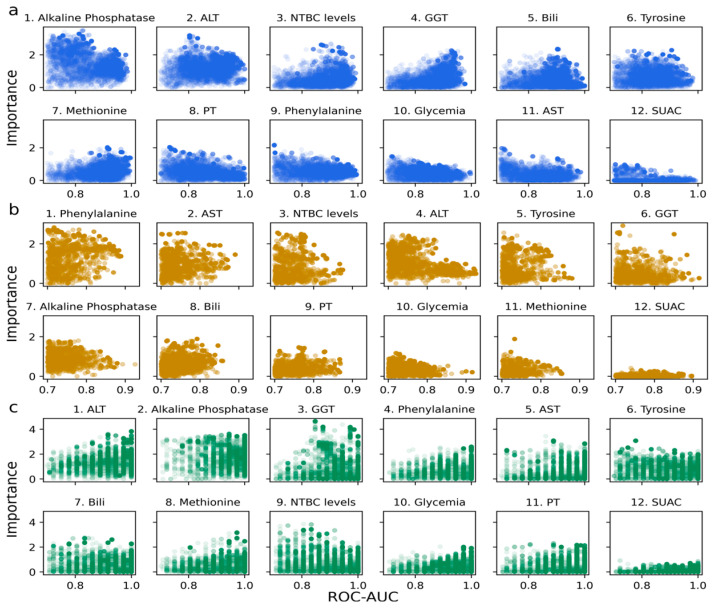
Model performance and feature importance. The importance of each variable is presented as a function of model performance for 5000 models per cohort. (**a**) cohort from Chile. (**b**) cohort from Rome. (**c**) cohort from Florence. Only models presenting ROC-AUC > 0.7 in testing are considered and shown.

Figure 2 presents the ML models with a testing performance ROC-AUC > 0.7 and the importance of the biochemical variables for each cohort. Here, each circle in the subplots represents a model associated with a specific ROC-AUC value and the importance level of the variable. This analysis enabled us to distinguish variables that exert a more significant influence than others in predicting altered AFP, resulting in a ranking of variable importance for each cohort separately. The subplots indicate the ranking position next to each variable’s name (Figure 2). 

**Figure 3:**
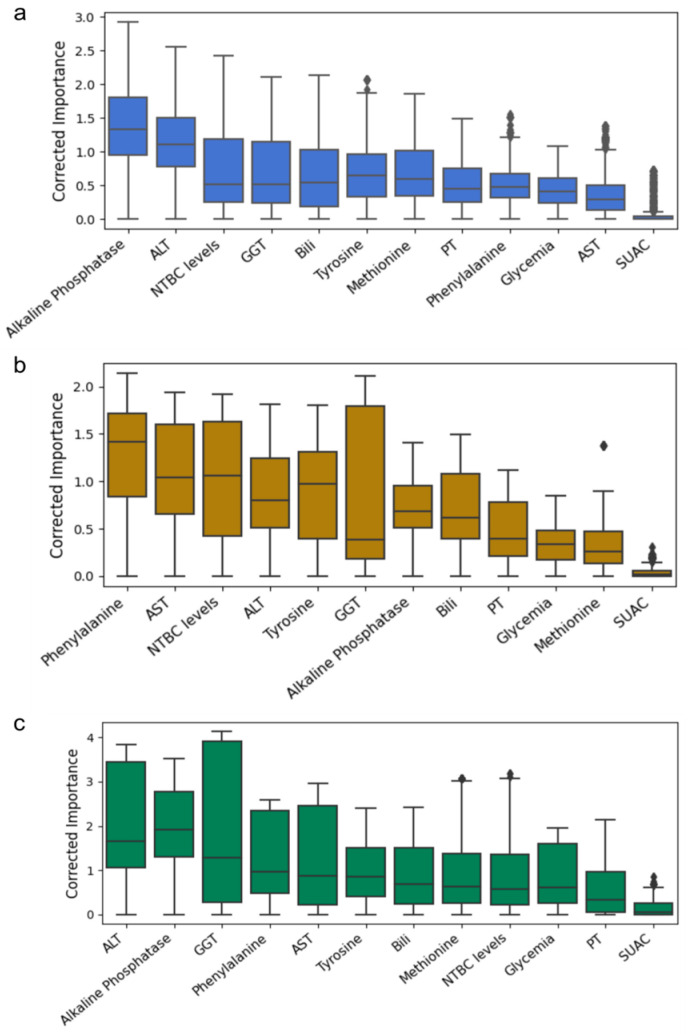
Variable Importance Ranking (VIR) for detecting altered levels of AFP. (**a**) VIR for Chile cohort. (**b**) VIR for Rome cohort. (**c**) VIR for Florence cohort. Corrected importance was calculated as the product between the importance and the ROC-AUC averaged across all models > 0.7. Supplementary Tables S3–S5 show statistical differences between variables.

We determined the significance of each variable by combining its importance and the performance of each model. This was achieved by computing the average of “corrected importance” for every variable; this resulting metric, termed “corrected importance”, derived from multiplying the variable’s significance (quantified using the Shapley value) by the ROC-AUC of its corresponding model. Figure 3 depicts the rankings based on the corrected importance, which varied slightly throughout the cohorts, with transaminases consistently ranked among the top five variables in three cohorts. Conversely, SUAC ranked last across all three cohorts (the statistical analysis of the corrected importance distribution difference is presented in Supplementary Tables S3–S5).

### 2.4. Feature Importance Based on Biochemical and Age-Related Variables

**Figure 4:**
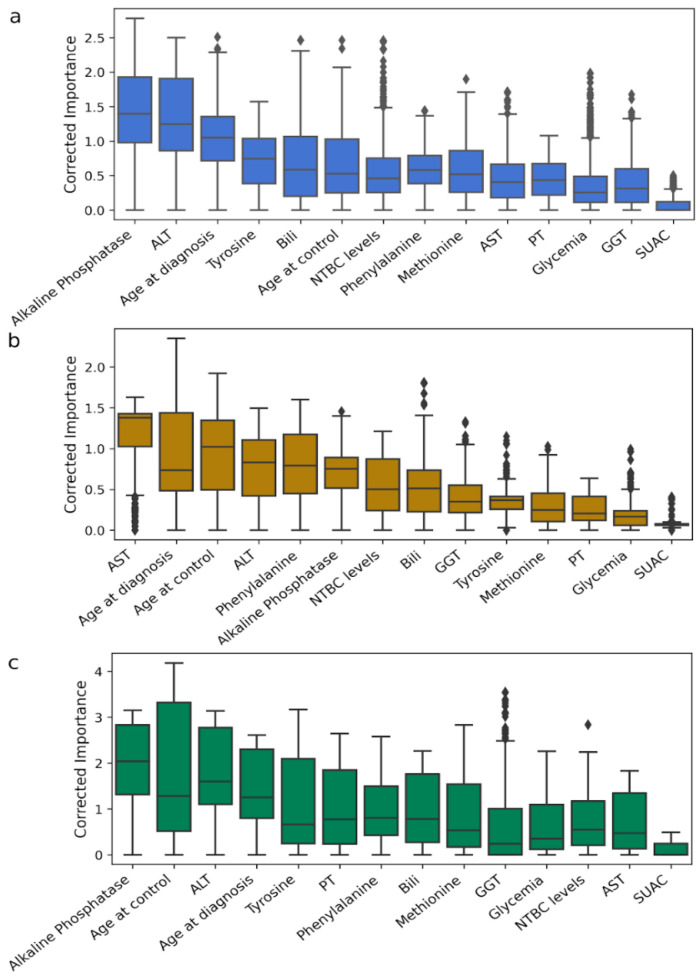
Variable Importance Ranking, including age-related variables. Variables are ranked according to their corrected importance in predicting altered levels of AFP. (**a**) VIR for Chile cohort. (**b**) VIR for Rome cohort. (**c**) VIR for Florence cohort. Supplementary Tables S6–S8 showed statistical differences between variables, while the dispersion of models can be found in Supplementary Figure S1.

The ML model procedure was repeated, including the age at diagnosis and age at control variables (Supplementary Figure S1), as these are identified as significant risk factors for HCC development. Figure 4 illustrates the resultant rankings, wherein age-related variables are positioned within the upper half. In the Chilean cohort, age at diagnosis and age at control were ranked third and sixth, respectively (Figure 4a). In the Roman cohort, age at diagnosis and age at control occupied the first and second positions, respectively (Figure 4b). For the Florentine cohort, age at control and age at diagnosis were ranked second and fourth, respectively (Figure 4c).

These results show that while biochemical markers remain crucial, age-related variables also seem to have an influence on determining AFP levels. Identifying these variables suggests that the ML model has captured clinically relevant information; enhanced phenotypic understanding; and revealed the complex, multifaceted nature of AFP alteration.

### 2.5. Identification of Key Variables Through Cross-Cohort Consensus Clustering

**Figure 5:**
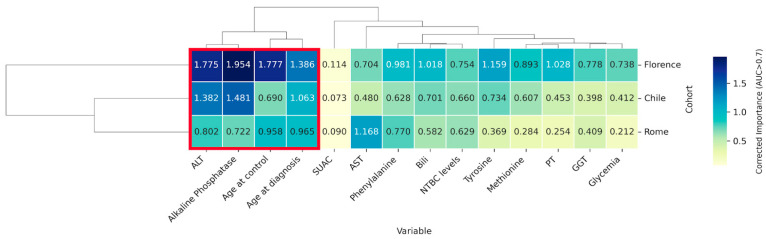
Hierarchical clustering of cohorts considering biochemical and age-related variables. Based on their corrected importance, clusters of variables and cohorts were determined. The average is shown inside each cell with color intensity proportional to its magnitude. The red square highlights the cluster comprising the most critical variables across cohorts.

The global pattern of common variables across cohorts suggests the existence of a core set of highly significant variables. We employed unsupervised hierarchical clustering analysis to identify this group of consensus variables. Figure 5 shows the clustering outcome, where a red square marks the cluster of highly important variables. Remarkably, this cluster includes age-related variables along with ALT and alkaline phosphatase. To further explore whether the same biochemical markers were still relevant without the influence of age variables in the predictive model, we conducted an additional exploratory clustering analysis excluding age-related variables (Supplementary Figure S2). Interestingly, even in the absence of age, ALT and alkaline phosphatase continued to form the most prominent cluster.

These four significant variables were selected for a logistic regression analysis to evaluate the performance and accuracy of combining these variables to detect elevated AFP values. As shown in Table 2, the model’s performance using all four variables presented an AUC of 0.800 for the testing instance. By iteratively omitting one variable at a time from the analysis, we assessed the impact of each variable individually within the model. Thus, we found that ALT removal had the most negative effect on the model’s testing performance for detecting elevated AFP levels (0.6745), suggesting that ALT is the most relevant predictor in the model.

### 2.6. AFP and ALT as Combined Biomarkers to Predict the Risk of Hepatocellular Carcinoma in HT-1 Patients

**Figure 6:**
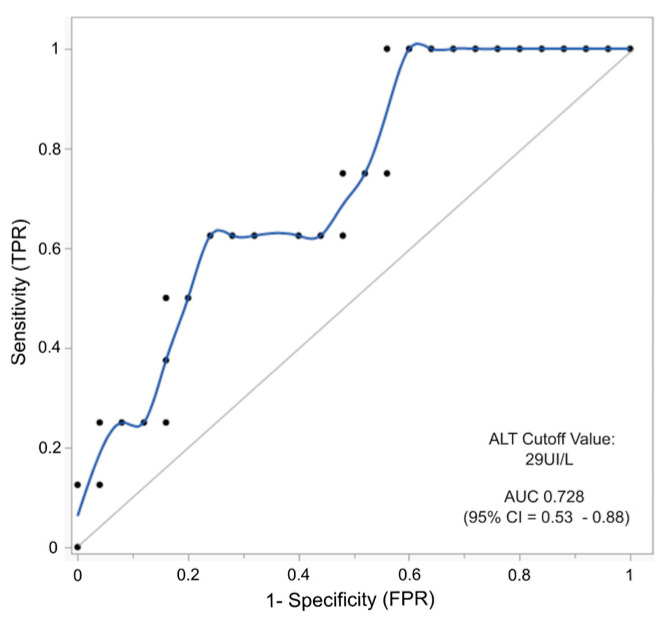
ALT cut-off value for HCC risk assessment. The area under the receiver operating characteristic (AUROC) curve is graphically represented to determine the optimal cut-off value of ALT for discriminating HCC risk. The model was constructed using mean ALT values for each patient (n = 33), categorized by whether they developed HCC (n = 8) or not (n = 25).

To evaluate the association between ALT (alanine transaminase) and HCC (hepatocellular carcinoma) in the context of HT-1, a receiver operating characteristic curve (ROC) analysis was performed to determine an optimal cut-off value for ALT in predicting HCC. Using the mean of all data records per patient (n = 32), both for those who developed HCC and those who did not, the ALT cut-off value calculated was 29 UI/L as the threshold for discriminating patients at risk of developing HCC. The performance of this model yielded an AUC of 0.73 (95% CI = 0.53–0.88) with a sensitivity of 1 and a specificity of 0.56, as shown in Figure 6. We then analyzed the mean AFP and ALT values for patients who developed HCC and those who did not (Table S9). Using these data, we established the percentage of true- and false-positive cases in our cohort by applying combined cut-off values for AFP and ALT. Using an AFP cut-off of 5 ng/mL alone, 52% of patients without HCC had levels above these limits. This proportion is reduced to 32% using combined cut-offs (AFP 5 ng/mL and ALT 29 UI/L). A similar reduction in false positives is achieved for the 10 ng/mL AFP cut-off, where 32% is reduced to 20% when both liver biomarkers are used. This combination approach reduced false positives, enhancing the diagnostic accuracy for HCC. Concerning sensitivity, no major improvement is observed when both biomarkers are used together, as seven out of eight patients (87.5%) who developed HCC presented levels above 5 ng/mL of AFP and >29 UI/L of ALT. Interestingly, the only patient who developed HCC with a low AFP value (<5 ng/mL) showed an elevated ALT level (38 UI/L) and an age at diagnosis of 36 months.

## 3. Discussion

NTBC-treated HT-1 patients are still at risk of developing severe liver complications, such as acute liver failure and hepatocellular carcinoma. This risk is particularly elevated in patients whose diagnosis and initiation of treatment are delayed beyond the newborn period. We employed machine learning (ML) techniques to perform in-depth data analysis on 13 routinely measured laboratory parameters from HT-1 patients in follow-up across three independent cohorts, aiming to identify which of them most significantly contributes to predicting elevated levels of AFP, a crucial biomarker of HCC.

Datasets commonly analyzed with ML in inborn errors of metabolism, such as metabolomics and newborn screening, often present high volumes of data [19,20,21,22,23]. In contrast, diseases such as HT-1 face the challenge of data scarcity [24,25]. Our ML approach demonstrates robustness and the capacity to derive insights even when training limited patient data.

The initial ML analysis integrating check-up data from urine, plasma, and dried blood samples, along with demographic information, provided a global overview of the similarities between the three cohorts, as we were unable to cluster them independently. This observation underscores that while the cohorts exhibit significant differences in key aspects, such as the age of diagnosis, age at the control, and NTBC dose (Table S1), the overall analysis of variables suggests that all three cohorts exhibit similar behavior. This commonality may be attributed to populations of patients under high surveillance and receiving appropriate treatment and management.

We considered an AFP threshold of 5 ng/mL in our supervised ML models to predict AFP alterations. Although AFP combined with liver imaging remains a key biomarker for HCC diagnosis and monitoring, its specificity and sensitivity for the early-stage detection of HCC are considered suboptimal, with elevated levels detected in only 60–80% of HCC cases [15,28,29,30,31]. The current AFP cut-off for HT-1 clinical monitoring is 10 ng/mL, which is manageable for patients, mainly those diagnosed late, where stabilizing AFP levels is challenging [12]. Choi et al.’s recent longitudinal study advised lowering the AFP threshold to 5 ng/mL to enhance early HCC detection sensitivity in at-risk patients [14]. Our cohort’s median AFP value showed that neonates diagnosed with low HCC risk had a median of 2.2 ng/mL (Florence cohort), similar to the findings of Couce et al. in a Spanish HT-1 cohort diagnosed by NBS [32]. Interestingly, recent studies have reported exceptional cases in which patients clinically diagnosed with HCC had remarkably low AFP levels, as low as 1.3 ng/mL [17]. In the Rome cohort, one patient developed HCC, and another had hepatoblastoma with an AFP level below 5 ng/mL. Thus, setting 5 ng/mL as the cut-off for AFP allowed us to identify four key predictive variables: two demographic and two laboratory biomarkers in an HT-1 population where 83% were diagnosed based on clinical manifestation. The patient’s age and the age at diagnosis were both identified as relevant. Notably, the age at diagnosis is one of the most critical factors that has been identified so far as a contributing predisposing factor to liver cancer development, increasing the risk of HCC 2.5-fold in patients who started the treatment within 1–6 months of age compared to those who started within the first month of life [33]. Hence, identifying this variable as one of the most influential in AI-based data analysis validates the performance of the models developed for predicting AFP levels. Transaminase ALT and alkaline phosphatase also emerged as key parameters influencing AFP levels. This discovery remained consistent across the three distinct cohorts from Chile and Italy, underscoring its significance across diverse demographic contexts. Notably, SUAC was not a significant factor in detecting AFP alterations despite its recognized value in assessing patient adherence to pharmacological treatment [4,31]. This result may be partially explained by the conversion of SUAC values into a binary classification (normal/elevated), which could have reduced the model’s sensitivity to more subtle variations. Moreover, SUAC levels are often below the detection limit in patients with good adherence, limiting the model’s variability and potentially introducing bias. Nevertheless, the low ranking of SUAC across cohorts, despite its pathognomonic role, underscores a key clinical insight: even with good treatment adherence and SUAC control, the risk of hepatic complications, including HCC, may persist in patients who initiate treatment late.

Comparing the results of the statistical methods and ML analyses reveals notable differences and complementary insights. Alkaline phosphatase was the most relevant variable for the Chilean and Florence cohorts, with higher corrected importance in the first and second ML analyses. This finding was not entirely revealed in the initial association analysis (Table 1), where a significant but weak correlation between alkaline phosphatase and AFP was found. While the transaminases ALT and AST were the variables that correlated most strongly with the AFP level, a post-ML hierarchical clustering analysis of cohort variables showed that only ALT was identified as a relevant predictor. Results from statistical methods and machine learning (ML) algorithms may not fully converge, which is expected [34]. ML techniques outperform traditional statistical analyses in identifying nonlinear relationships between variables—relationships that might be missed in classical univariate association analysis [34]. This ability enables ML to uncover patterns and insights that are not easily discernible through conventional statistical methods, providing a more profound understanding of complex datasets [35,36]. However, the insights derived from statistical methods and machine learning (ML) algorithms are complementary, offering a more complete and balanced approach to data analysis.

We observed that removing the ALT variable from a logistic regression model built with the four most essential variables greatly impacted the model’s predictive power to detect altered AFP levels, lowering the AUC from 0.800 to 0.6745. This finding suggests that ALT plays the most critical role in identifying patients with altered AFP levels compared to the other variables included in the model. ALT and alkaline phosphatase are not direct markers of liver function but indicators of liver cell injury and biliary tract disruption. Elevated levels of these enzymes can signify various forms of liver damage or underlying hepatic diseases, including HCC. In particular, alkaline phosphatase is an important biomarker for skeletal metabolism and disease. Hence, alterations in its concentration deserve a careful clinical interpretation, considering the patient’s age and sex [37]. While the relationship between ALT elevations and HCC is well established in chronic liver diseases, its specific role as a biomarker for HCC in HT-1 patients has been minimally considered [38]. Nonetheless, persistent or fluctuating elevations of these enzymes, particularly ALT, may serve as valuable early indicators of hepatic stress or evolving pathologies in HT-1 patients, potentially preceding more specific markers of HCC development. We explored this issue by assessing the predictive potential of ALT for HCC risk through an AUROC analysis. Given the limited sample size, this AUROC analysis should be interpreted with caution. While the model yielded a cut-off value of 29 UI/L for ALT, associated with an AUC of 0.73 (95% CI: 0.53–0.88), the broad confidence interval reflects the exploratory nature of this finding and underscores the need for further validation. Notably, the identified threshold lies within what is typically considered the normal reference range for ALT—values that themselves can vary depending on the characteristics of the population studied and the presence of risk factors for liver disease [38,39]. Despite the widespread use and standardization of ALT for liver disease screening, such as in Non-Alcoholic Fatty Liver Disease (NAFLD), there is no consensus on the upper limits of normal ALT levels in children. Several studies have proposed sex-specific cut-offs. In the United States, cut-offs of 22 UI/L for females and 26 UI/L for males have been validated in diverse cohorts. A Canadian study suggested an upper limit of 30 UI/L for children aged 1 to 12 years and 24 UI/L for those between 13 and 19 years [40,41]. The ALT mean values in our cohort of patients showed that the Florence cohort is the only group that presented a mean value below 29 UI/L, which is consistent with their early diagnosis through newborn screening; maintained AFP levels below 5 ng/mL; and had a low risk of developing HCC. While the presence of HCC can lead to increased ALT levels, it is fundamental to recognize that elevated ALT levels alone are not a definitive indicator of HCC. However, combining the cut-off values for both ALT and AFP may improve the specificity of AFP compared to using AFP alone, regardless of whether we used an AFP cut-off of 5 or 10 ng/mL. Also, the sensibility could be improved if we also consider the age at diagnosis, as in the case of a single HCC-positive patient presenting low levels of AFP, higher levels of ALT, and advanced age when starting the treatment (36 months).

Our latest findings suggest a promising approach for enhancing HCC surveillance in HT-1 patients. Identifying routinely used and widely accessible laboratory markers, particularly ALT levels, as predictors of altered AFP could enhance our ability to stratify risk and tailor monitoring protocols, mainly in patients with major risk due to late diagnosis. However, it is crucial to acknowledge that the limited sample size of this cohort and the lack of temporal alignment in data collection across patients represent a significant limitation. More extensive longitudinal prospective studies are needed to validate these findings and determine HCC risk using the identified biomarkers and age factors. Such comprehensive research would confirm the clinical significance of our findings and assess their reproducibility across diverse patient populations, accounting for variations in genetic backgrounds, treatment regimens, and environmental factors. Furthermore, it would allow for the exploration of age and sex-specific thresholds, potentially leading to more personalized surveillance strategies.

This study consistently reinforces the importance of neonatal screening for tyrosinemia type 1 disease. Early pharmacological treatment and nutritional guidance can prevent later-life clinical complications, improving the patient’s quality of life. Additionally, these approaches offer socioeconomic benefits by reducing medical costs associated with managing acute liver failure or treating liver cancer.

## 4. Materials and Methods

### 4.1. Patient Cohort and Eligibility Criteria

The research protocol and study design involve a longitudinal retrospective analysis of clinical and biochemical data records collected during clinical outpatient controls at three local reference centers for HT-1 follow-up: Institute of Nutrition and Food Technology from the University of Chile, Santiago, Chile; the Ospedale Pediatrico Bambino Gesú IRCCS (OPBG) in Rome Italy; and Meyer’s Hospital in Florence, Italy. Clinical records from Chilean patients were collected from 2019 to 2023 during quarterly control visits, as mandated by the Health Minister of Chile under the financial support for the high-cost drugs system (Law 20.500, Ricarte Soto). Rome’s patient records cover the period from 2015 to 2022, and Florence’s records span 2011 to 2023. Tyrosinemia type 1 patients, confirmed by clinical and biochemical criteria according to international consensus guidelines [2,4], were included in the analysis, regardless of whether they were diagnosed via clinical manifestation or newborn screening. In the Chilean cohort, almost all patients were diagnosed because of symptomatic manifestation, and only one was diagnosed during the newborn period owing to familiar antecedents. Patients from Rome were all diagnosed late based on clinical symptoms, while in the Florence cohort, patients were all diagnosed during newborn screening. For patients who developed HCC and underwent liver transplantation (LTx), only records before the transplant were taken into consideration. The total number of patients was 35, 20 from Chile (2 with HCC and LTx), 10 from OPBG (6 with HCC and LTx), and 5 from Meyer’s Hospital in Florence. Thirteen biochemical, pharmacological, and metabolic biomarker variables were selected based on their commonality across the three medical centers (Table S1). This choice comes from the shared analytical and methodological procedures undertaken, the panel of biochemical parameters evaluated in each control, and the nutritional and pharmacological management of the patients following the consensus guidelines for the metabolic monitoring of HT-1 patients [2,4]. Additionally, two demographic variables—age at the moment of diagnosis and age at the moment of the record (current age)—were included because of their identified role as risk factors for complications in HT-1, as described by Mayorandan [33].

### 4.2. Dataset and Statistical Analysis

The retrospective longitudinal dataset included 135, 56, and 38 independent clinical records from patients from Chile, Rome, and Florence centers. Each institutional ethics committee approved the clinical research protocol. We included 220 outpatient records for the ML analysis. Each record had to contain at least 70% of the variables and include data from both AFP and SUAC measurements. SUAC data were categorized as detected/not detected in urine or dried blood spot (DBS) samples. AFP was the binary target variable (normal: <5 ng/mL; altered: >5 ng/mL). Biochemical variables included NTBC levels in DBS; AFP levels; plasma levels of phenylalanine (Phe), methionine (Met), and tyrosine (Tyr); ALT; AST; GGT; prothrombin time (PT); total bilirubin (Bili); alkaline phosphatase; and glycemia. Statistical analysis included the Anderson–Darling test for normality, the Kruskal–Wallis with Dunn’s post hoc tests for group comparisons, and Spearman’s correlation for abnormal data. Significance was set at *p* < 0.05 using the JMP^®^ Pro 18.0.2, JMP Statistical Discovery LLC.

### 4.3. Missing Data Handling and Unsupervised Analysis

The percentages of missing values in each cohort were 3.05%, 4.62%, and 7.07% for Chile, Rome, and Florence, respectively. The missing data were imputed using the Iterative Imputer method from Python’s Scikit-learn library (version 1.5) [42] implemented in Python version 3.9 (Python Software Foundation, 2020) and based on Multivariate Imputation by Chained Equations [43]. Nonlinear dimensionality reduction was performed with Uniform Manifold Approximation and Projection (UMAP) using umap-learn by Python version 3.9 (Python Software Foundation, 2020) [44].

### 4.4. Predictive Model

A binary classification of normal or altered levels was established to predict elevated AFP levels based on whether a value was <5 ng/mL or >5 ng/mL, respectively. We employed the well-established eXtreme Gradient Boosting (XGBoost) algorithm [45], a decision-tree-based ensemble machine learning technique for its proven effectiveness in biomedicine and biomarker discovery [25,46,47]. The Python implementation from the XGBoost package version 3.0 was used

The models were trained exclusively using 70% of the Chilean cohort dataset, while the remaining 30% of the Chilean cohort, along with the Rome and Florence cohorts, served as test datasets. A strict separation between training and testing subsets from the Chile cohort was maintained to ensure the algorithm’s validation. It did not combine them or use the same patient data employed during the training. Thus, the 30% testing Chilean subset was never used during the model training phase. This approach prevents data leakage and ensures that the model’s performance metrics reflect its true predictive capabilities. Testing the model with the external datasets from Rome and Florence further validated the robustness of the predictions, indicating that our findings apply to the Chilean population and are generalizable across different cohorts.

The performance of the models was assessed in the testing stage using the area under the receiver operating characteristic curve (AUROC) [48]. To enhance model interpretability, we utilized SHAP (Shapley Additive exPlanations) values, which, based on game theory, offer a detailed understanding of how each feature in the dataset influences the model’s predictions [49], revealing their importance in the predictive model.

We calculated a “corrected importance” by multiplying the testing performance (AUROC) by variable importance (SHAP value) and then averaged these values to create a comprehensive ranking of variable importance.

### 4.5. Multi-Model Approach for Robust Generalization and Explainability

We used a multimodal approach consistent with our previous methodology [25]. This approach explored several instances of a model using a framework for automated hyperparameter optimization. This method enables the creation of multiple machine-learning models by training on different bootstrapped datasets. For each new model training instance (or “independent training rounds”), we generated a new train/test partition from the Chilean cohort, maintaining a consistent 70:30 split. While the training and test fractions remained separate within each training instance and were never combined, they varied across different model iterations (see method flowchart in Figure A1). This method allowed us to sample multiple models with different testing performance metrics and feature explainability profiles [25]. Here, the main objective was to fully encompass the wide range of possible models generated from our data displaying diverse performance levels and rankings of variable importance. This allowed us to selectively focus on the high-performing models to ensure a robust generalization of the phenotype. Combining these models enhances the predictive capabilities. The inherent randomness and diversity introduced through bootstrapping allow the models to capture varied data perspectives and mitigate overfitting risks. Each model in our study was created and evaluated based on hyperparameter optimization. Unlike parameters learned from the data, hyperparameters are set before the learning process begins. We dynamically adjusted the hyperparameters to sample a diverse set of models. We created several XGBoost instances by varying the depth of the tree in the model, the learning rate, the fraction of the training data to be randomly sampled for each tree, L2 regularization, L1 regularization, the scaling factor for the gradient for the positive class (balancing the positive and negative weights), and the number of folds in the K-fold cross-validation. For each sampled model, we assessed two key aspects: the model’s ability to distinguish between normal and elevated AFP, quantified by the AUROC, and the model’s explainability, determined using SHAP values. Our method involved running multiple repetitions of this process (5000), each time sampling different models.

## Figures and Tables

**Table 1:** Association of AFP levels with biochemical, metabolic, and demographic variables.

Variable	Spearman’s ρ	*p*-Value	N° of Samples
ALT	0.534	<0.0001	228
AST	0.509	<0.0001	223
Age at Diagnosis	0.4973	<0.0001	231
GGT	0.449	<0.0001	211
Prothrombin Time	0.299	0.0002	206
Alkaline Phosphatase	0.297	<0.0001	200
Total Billirrubin	0.208	0.0034	196
Methionine	0.0853	0.216	212
Glycemia	−0.0328	0.627	222
Age at Control	−0.0598	0.3796	218
NTBC Levels	−0.052	0.4456	217
Phenylalanine	−0.1204	0.0818	210
Tyrosine	−0.149	0.0298	212

**Table 2:** Logistic regression model for predicting altered AFP. The ROC-AUC values obtained in each instance (training, validation, and testing) of the logistic regression model are indicated for the five models built with the four most important variables identified by the ML analysis. Each “model–variable” curve indicates that the specified variable was omitted from the original model, allowing for the assessment of its impact on predictive performance.

	AUROC		
Logistic Regression Models	Training	Validation	Testing
Model–ALT	0.6954	0.5625	0.6745
Model–ALKP	0.7722	0.5463	0.7993
Model–Age	0.7909	0.5208	0.8246
Model–Age at Diagnosis	0.7892	0.5677	0.7833
Complete Model	0.8157	0.6563	0.8000

## Data Availability

The datasets presented in this article are not readily available because the data are part of an ongoing study. Requests to access the datasets should be directed to the corresponding author.

## Supplementary Materials

The supporting information can be downloaded at https://www.mdpi.com/article/10.3390/ijms26083839/s1.

## Abbreviations

The following abbreviations are used in this manuscript:

AFP	Alpha-fetoprotein
ALT	Alanine aminotransferase
AST	Aspartate aminotransferase
AUC	Area under the curve
Bili	Total bilirubin
DBS	Dried blood spot
GGT	Gamma-glutamyl transferase
HCC	Hepatocellular carcinoma
HT-1	Hereditary tyrosinemia type 1
Met	Methionine
ML	Machine learning
NBS	Newborn screening
NTBC	Nitisinone
Phe	Phenylalanine
PT	Prothrombin time
ROC	Receiver operating characteristic curve
Tyr	Tyrosine
SUAC	Succinylacetone
