# Supplementary material for: Integrating Machine Learning and Follow-Up Variables to Improve Early Detection of Hepatocellular Carcinoma in Tyrosinemia Type 1: A Multicenter Study
Source: Int J Mol Sci. 2025 Apr 18;26(8):3839. doi: 10.3390/ijms26083839 (PMC12028188; doi:10.3390/ijms26083839)
Supplement: Supplementary file 1 [file ijms-26-03839-s001.zip › ijms-3542912-supplementary.pdf]

SUPPLEMENTARY INFORMATION

Title:

Integrating Machine Learning and Follow-up Variables to Improve Early Detection of Hepatocellular Carcinoma in Tyrosinemia Type-1: A Multicenter Study

Authors:

Karen Fuenzalida[1], María Jesús Leal-Witt[1], Alejandro Acevedo[1], Manuel Muñoz[1], Camila Gudenschwager[1], Carolina Arias[1], Juan Francisco Cabello[1], Giancarlo La Marca[2,3], Andrea Pietrobattista[4], Marco Spada[5], Cristiano Rizzo[4], Carlo Dionisi-Vici[4], Verónica Cornejo[1]

Table of contents:

- **Supplementary Figure S1**. Model performance and feature importance, including age-related variables.
- **Supplementary Figure S2.** Hierarchical clustering of cohorts based on biochemical variables.
- **Supplementary Table S1**. Descriptive Statistics and Comparative Analysis of Variables Across the Three Cohorts
- **Supplementary Table S2.** Descriptive statistics of patients who presented and did not present HCC in Rome and Chile cohorts
- **Supplementary Table S3.** Biochemical variables of the Chilean cohort
- **Supplementary Table S4.** Biochemical variables of the Rome cohort
- **Supplementary Table S5.** Biochemical variables of the Florence cohort
- **Supplementary Table S6.** Biochemical and age-related variables of the Chilean cohort
- **Supplementary Table S7.** Biochemical and age-related variables of the Rome cohort
- **Supplementary Table S8.** Biochemical and age-related variables of the Florence cohort.
- **Supplementary table S9.** Comparison between HCC and non-HCC patients.

Supplementary Figure S1.

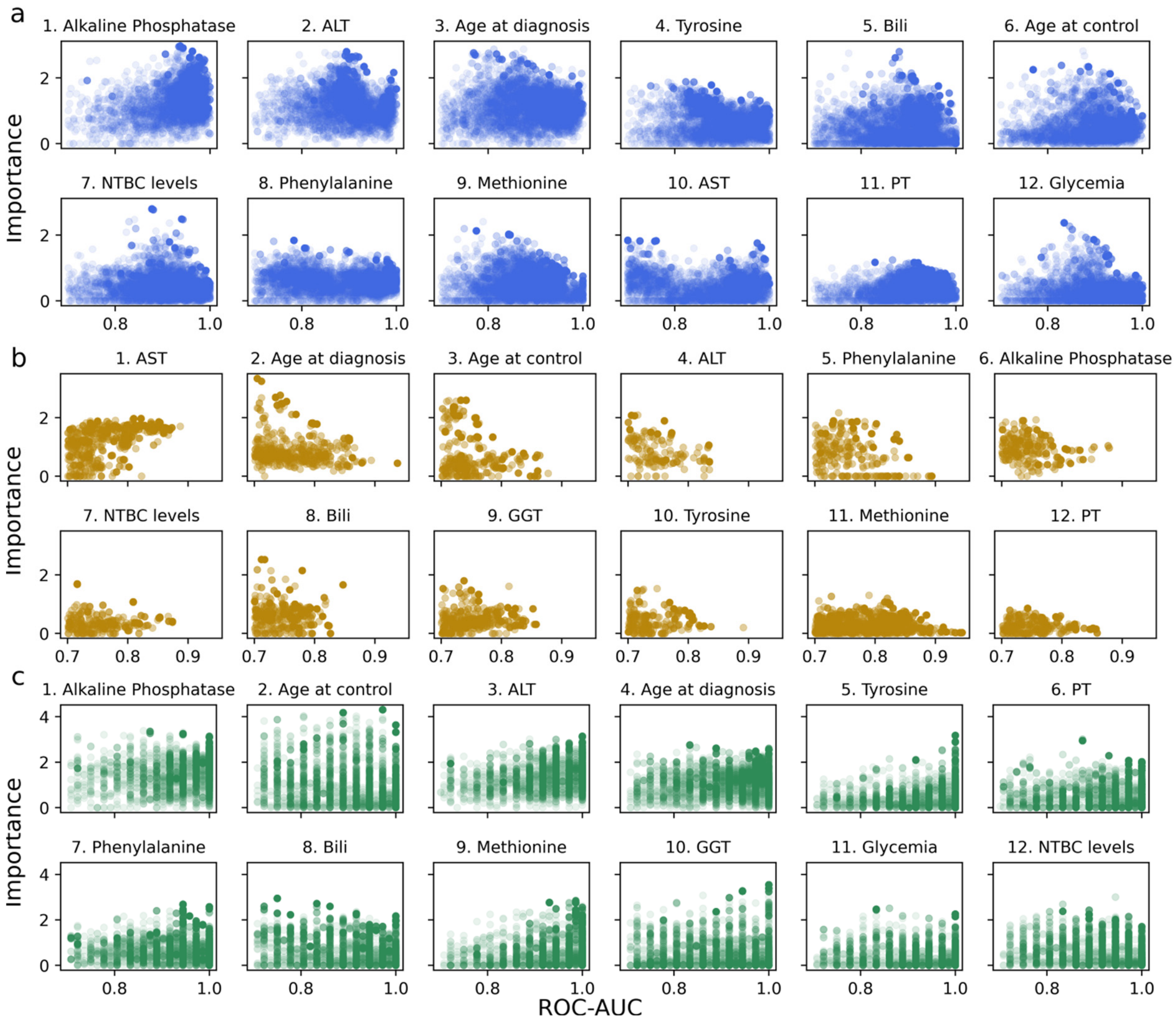

Supplementary Figure S1. Model performance and feature importance, including age-related variables. The importance of each variable is presented as a function of model test performance (ROC-AUC > 0.7) for 5000 models per cohort. Cohorts from Chile (a), Rome (b), and Florence (c) are shown. Variables are sorted from most to least important.

Supplementary Figure S2

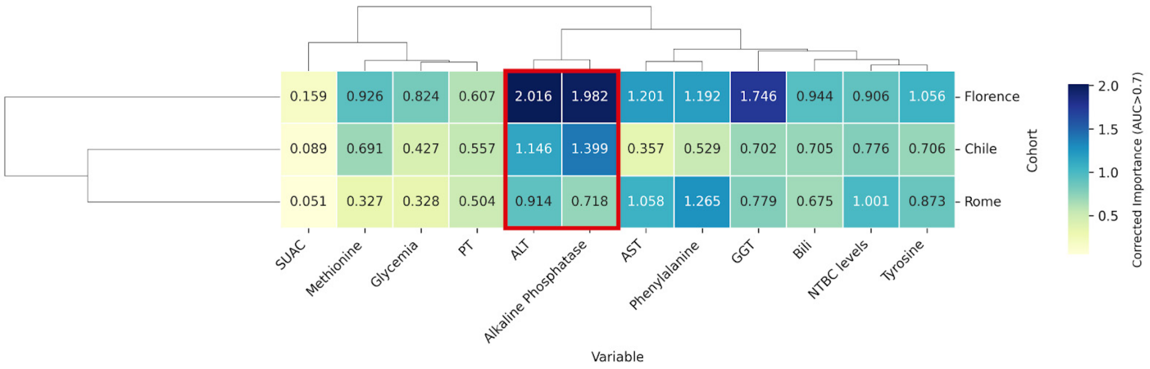

Supplementary Figure S2. Hierarchical clustering of cohorts based on biochemical variables. The red square highlights the cluster with the most significant variables across cohorts.

Supplementary Table S1.

Variable	Chile	Italy - Rome	Italy - Florence	p*	p¥	pϕ
NTBC Dosis (mg/kg/day)	0.93 ± 0.14	0,67 ± 0.14	1 ± 0.04	< 0.001	0.5	0.001
NTBC Levels (µmol/L)	21.7 ± 6.8	38.5 ± 11.5	26.2 ± 6.4	< 0.0001	0.49	< 0.05
Plasma Tyrosine (µmol/L)	456.3 (307–1040)	452 (130–908,5)	434 (401–852)	1	1	1
Plasma Phenylalanine (µmol/L)	46.8 ± 18.2	54,2 ± 15.6	44.6 ± 5.3	0.5	0.96	0.55
Plasma Methionine (µmol/L)	27.1 ± 5.5	25.9 ± 7.2	21.8 ± 1.2	0.87	0.17	0.42
Alfa-fetoprotein (ng/mL)	9.2 (1.3–505)	39.6 (2.3–3467)	2.2 (1.7–4.4)	1	< 0.05	< 0.05
Prothrombin time (sec)	13.9 ± 1	14.3 ± 0.9	10.6 ± 1	0.65	<0.0001	< 0.0001
Total Billirrubin (UI/L)	0.46 (0.28–1.59)	0.45 (0.26–1.6)	0.3 (0.2–0.35)	1	0.08	<0.05
ALT (GPT UI/L)	35 ± 16.6	45.3 ± 35	28.3 ± 7	0.5	0.8	0.37
AST (GOT UI/L)	42.5 ± 18.3	40 ± 16.2	34.6 ± 10.3	0.96	0.63	0.78
GGT (UI/L)	50.2 ± 34.6	69 ± 104	9.1 ± 3.2	0.72	0.41	0.2
Alkaline Phosphatase (UI/L)	298 ± 137	594 ± 363	284 ± 39.4	<0.05	0.99	< 0.01
Glycemia (mg/dL)	84.4 ± 5.7	87.8 ± 5.6	81.3 ± 7.2	0.42	0.46	0.13
Age at diagnosis (months)	9 (1–63)	20 (6–48)	0.2 (0.2–0.33)	0.37	< 0.01	< 0.001
Age at control (years)	7.6 (1–23.2)	12.3 (3.9–24.8)	4.5 (3.4–7.9)	0.95	0.21	0.07
Sex (male %)	45	40	100			

Supplementary Table S1. Descriptive Statistics and Comparative Analysis of Variables Across the Three Cohorts. Data are presented as median (minimum and maximum) or mean ± standard deviation, depending on their distribution. Statistical comparisons were performed using the Kruskal-Wallis test followed by Dunn's multiple comparison test for non-parametric data or one-way ANOVA followed by Tukey's test for parametric data. Significant differences ($p < 0.05$) are highlighted in red: * Chile vs. Rome; ¥ Chile vs. Florence, and ϕ Florence vs. Rome. Mean values for each variable were first calculated per patient. Cohort sizes: Chile (n=20), Rome (n=6), and Florence (n=5).

Supplementary Table S2

	Chile		Italy - Rome	
**Variable**	**- HCC (n=18)**	**+ HCC (n=2)**	**- HCC (n=4)**	**+ HCC (n=6)**
NTBC Dosis (mg/kg/day)	0.92 ± 0.15	1 ± 0.08	0.68 ± 0.1	0.66 ± 0.3
NTBC Levels (μmol/L)	21.6 ± 7.2	22 ± 0.9	40.1 ± 13	35 ± 11
Plasma Tyrosine (μmol/L)	456 (307–1040)	462 (322–603)	662.4 (452–908)	442 (130.3–883)
Plasma Phenylalanine (μmol/L)	46.2 ± 17.6	52.4 ± 31	60 ± 22	49.6 ± 8
Plasma Methionine (μmol/L)	26.1 ± 4.3	35.3 ± 10	24.2 ± 4.8	27.2 ± 9.1
Alfa-fetoprotein (ng/mL)	8.6 (1.3–69.9)	1698 (178–3218)	3.11 (2.3–12.4)	180 (2.92–3467)
Prothrombin time (sec)	13.9 ± 1	13.5 ± 0.03	14.2 ± 1.2	14.3 ± 0.7
Total Billirrubin (UI/L)	0.56 ± 0.3	0.28 ± 0.01	0.49 ± 0.23	1.07 ± 0.62
ALT (GPT UI/L)	34.2 ± 17	42.7 ± 15	32.9 ± 10.4	53.6 ± 44
AST (GOT UI/L)	41 ± 17.8	55.9 ± 23	32.8 ± 13	46.3 ± 17
GGT (UI/L)	47.5 ± 30.3	71.1 ± 53.2	21 ± 15.3	101 ± 128
Alkaline Phosphatase (UI/L)	284 ± 138	406 ± 88	545 ± 508	627 ± 281
Glycemia (mg/dL)	84.9 ± 5.8	83.9 ± 7.5	86.5 ± 3.2	88.8 ± 7.2
Age at diagnosis (months)	7.5 (1–63)	19 (12–26)	13 (6–31)	29 (12–48)
Age at control (years)	9.2 (2.7–23.2)	6 (4.9–7)	12.3 (3.9–24.2)	16.6 (8.4–24.8)
Sex (male %)	38.9	100	50	33.3

Supplementary Table S2. Descriptive statistics of patients who presented and did not present HCC in Rome and Chile cohorts. Data are presented as median (minimum and maximum) or mean ± standard deviation, depending on their distribution (normal or abnormal).

Supplementary Table S3

	Alkaline Phosphatase	ALT	NTBC levels	GGT	Billi	Tyr	Met	PT	Phe	Glycemia	AST
Alkaline Phosphatase											
ALT	***										
NTBC levels	***	***									
GGT	***	***	*								
Bili	***	***	***	**							
Tyrosine	***	***	*	***	***						
Methionine	***	***	ns	***	***	ns					
PT	***	***	***	***	***	***	***				
Phenylalanine	***	***	***	***	***	***	***	ns			
Glycemia	***	***	***	***	***	***	***	***	***		
AST	***	***	***	***	***	***	***	***	***	***	
SUAC	***	***	***	***	***	***	***	***	***	***	***

Supplementary Table S3. Biochemical variables of the Chilean cohort. Statistical comparisons between the distributions of the corrected importance of biochemical variables are presented in Figure 3a. Kruskal–Wallis test- Dunn's post-test and FDR adjustment was performed, *: p-value < 0.05, **: p-value < 0.01, *** p < 0.001.

Supplementary Table S4

	Phe	AST	NTBC levels	ALT	Tyr	GGT	Alkaline Phosphatase	Billi	PT	Glycemia	Met
Phenylalanine											
AST	*										
NTBC levels	***	ns									
ALT	***	*	ns								
Tyrosine	***	*	ns	ns							
GGT	***	***	***	***	**						
Alkaline Phosphatase	***	***	*	ns	ns	ns					
Bili	***	***	***	***	*	ns	ns				
PT	***	***	***	***	***	*	***	***			
Glycemia	***	***	***	***	***	***	***	***	**		
Methionine	***	***	***	***	***	***	***	***	**	ns	
SUAC	***	***	***	***	***	***	***	***	***	***	***

Supplementary Table S4. Biochemical variables of the Rome cohort. Statistical comparisons between the distributions of the corrected importance of biochemical variables are presented in Figure 3b. Kruskal–Wallis test- Dunn's post-test and FDR adjustment was performed, *: p-value < 0.05, **: p-value < 0.01, *** p < 0.001.

Supplementary Table S5

Variable	ALT	Alkaline Phosphatase	GGT	Phen	AST	Tyr	Billi	Met	NTBC levels	Glycemia	PT
ALT											
Alkaline Phosphatase	**										
GGT	***	***									
Phenylalanine	***	***	***								
AST	***	***	***	***							
Tyrosine	***	***	***	***	*						
Bili	***	***	***	***	***	***					
Methionine	***	***	***	***	***	***	ns				
NTBC levels	***	***	***	***	***	***	**	**			
Glycemia	***	***	***	***	***	***	**	**	ns		
PT	***	***	***	***	***	***	***	***	***	***	
SUAC	***	***	***	***	***	***	***	***	***	***	***

Supplementary Table S5. Biochemical variables of the Florence cohort. Statistical comparisons between the distributions of the corrected importance of biochemical variables are presented in Figure 3c. Kruskal–Wallis test- Dunn's post-test and FDR adjustment was performed, *: p-value < 0.05, **: p-value < 0.01, *** p < 0.001.

Supplementary Table S6

Variable	Alkaline Phosphatase	ALT	Age at diagnosis	Tyr	Bili	Age at control	NTBC levels	Phe	Met	AST	PT	Glycemia	GGT
Alkaline Phosphatase													
ALT	***												
Age at diagnosis	***	***											
Tyrosine	***	***	***										
Bili	***	***	***	***									
Age at control	***	***	***	***	ns								
NTBC levels	***	***	***	***	***	***							
Phenylalanine	***	***	***	***	ns	ns	***						
Methionine	***	***	***	***	***	***	ns	***					
AST	***	***	***	***	***	***	***	***	***				
PT	***	***	***	***	***	***	***	***	***	ns			
Glycemia	***	***	***	***	***	***	***	***	***	***	***		
GGT	***	***	***	***	***	***	***	***	***	***	***	*	
SUAC	***	***	***	***	***	***	***	***	***	***	***	***	***

Supplementary Table S6. Biochemical and age-related variables of the Chilean cohort. Statistical comparisons between the distributions of the corrected importance of biochemical variables are presented in Figure 4a. Kruskal–Wallis test- Dunn’s post-test and FDR adjustment was performed, *: p-value < 0.05, **: p-value < 0.01, *** p < 0.001.

Supplementary Table S7

Variable	AST	Age at diagnosis	Age at control	ALT	Phe	Alkaline Phosphatase	NTBC levels	Bili	GGT	Tyr	Met	PT	Glycemia
AST													
Age at diagnosis	ns												
Age at control	ns	ns											
ALT	ns	ns	ns										
Phenylalanine	ns	ns	ns	ns									
Alkaline Phosphatase	ns	ns	ns	ns	ns								
NTBC levels	**	ns	ns	ns	ns	ns							
Bili	**	ns	ns	ns	ns	ns	ns						
GGT	***	*	*	ns	ns	ns	ns	ns					
Tyrosine	***	*	*	ns	ns	ns	ns	ns	ns				
Methionine	***	***	***	*	*	*	ns	ns	ns	ns			
PT	***	***	***	*	*	*	ns	ns	ns	ns	ns		
Glycemia	***	***	***	**	**	**	*	*	ns	ns	ns	ns	
SUAC	***	***	***	***	***	***	***	***	**	**	*	ns	ns

Supplementary Table S7. Biochemical and age-related variables of the Rome cohort. Statistical comparisons between the distributions of the corrected importance of biochemical variables are presented in Figure 4b. Kruskal–Wallis test- Dunn's post-test and FDR adjustment was performed, *: p-value < 0.05, **: p-value < 0.01, *** p < 0.001.

Supplementary Table S8

Variable	Alkaline Phosphatase	Age at control	ALT	Age at diagnosis	Tyr	PT	Phe	Bili	Met	GGT	Glycemia	NTBC levels	AST
Alkaline Phosphatase													
Age at control	***												
ALT	***	***											
Age at diagnosis	***	ns	***										
Tyrosine	***	***	***	***									
PT	***	***	***	***	***								
Phenylalanine	***	***	***	***	ns	**							
Bili	***	***	***	***	***	ns	***						
Methionine	***	***	***	***	***	***	***	***					
GGT	***	***	***	***	***	***	***	***	***				
Glycemia	***	***	***	***	***	***	***	***	***	***			
NTBC levels	***	***	***	***	***	***	***	***	***	***	***		
AST	***	***	***	***	***	***	***	***	***	***	ns	***	
SUAC	***	***	***	***	***	***	***	***	***	***	***	***	***

Supplementary Table S8. Biochemical and age-related variables of the Florence cohort. Statistical comparisons between the distributions of the corrected importance of biochemical variables are presented in Figure 4c. Kruskal–Wallis test- Dunn's post-test and FDR adjustment was performed, *: p-value < 0.05, **: p-value < 0.01, *** p < 0.001.

Supplementary Table S9

Patient	Origen	HCC	AFP (ng/mL)	ALT (UI/L)	Age at Diagnosis (month)
P1	Chile	No	10.8	33.3	7
P2	Chile	No	31.9	49.7	25
P3	Chile	No	26.3	38.0	63
P4	Chile	No	9.0	19.3	3
P5	Chile	No	9.4	70.1	4
P6	Chile	No	7.7	45.8	11
P7	Chile	No	1.3	24.4	2
P8	Chile	No	6.5	13.8	6
P9	Chile	No	10.8	19.5	1
P10	Chile	No	3.7	18.0	3
P11	Chile	No	5.8	34.0	10
P12	Chile	No	17.3	34.0	15
P13	Chile	No	3.1	22.2	31
P14	Chile	No	8.2	23.4	4
P1	Florence	No	1.7	32.0	0.2
P1	Rome	No	2.3	19.3	18
P2	Florence	No	2.1	19.2	0.2
P3	Florence	No	4.4	35.8	0.3
P4	Florence	No	4.2	32.3	0.33
P5	Florence	No	2.2	22.1	0.2
P2	Rome	No	3.9	39.8	6
P3	Rome	No	12.4	42.0	31
P4	Rome	No	2.4	30.5	8
P15	Chile	No	24.0	28.0	4
P16	Chile	No	3.3	35.6	8
P17	Chile	Yes	3218.7	32.1	12
P5	Rome	Yes	605.0	29.0	48
P6	Rome	Yes	3467.0	142.7	18
P7	Rome	Yes	67.0	29.0	22
P8	Rome	Yes	2.9	38.0	36
P9	Rome	Yes	75.4	41.3	48
P18	Chile	Yes	178.9	53.3	26
P10	Rome	Yes	285.6	41.8	12

Supplementary Table S9. Comparison of risk factors between HCC and non-HCC patients. Summary of ALT, AFP mean values, and age at diagnosis for each patient from the Chilean and Italian cohorts. The patients are classified based on whether patients developed HCC or not.
